# Administration of Steamed and Freeze-Dried Mature Silkworm Larval Powder Prevents Hepatic Fibrosis and Hepatocellular Carcinogenesis by Blocking TGF-β/STAT3 Signaling Cascades in Rats

**DOI:** 10.3390/cells9030568

**Published:** 2020-02-28

**Authors:** Da-Young Lee, Sun-Mi Yun, Moon-Young Song, Sang-Deok Ji, Jong-Gon Son, Eun-Hee Kim

**Affiliations:** 1 College of Pharmacy and Institute of Pharmaceutical Sciences, CHA University, Seongnam 13488, Korea; angela8804@naver.com (D.-Y.L.); sun21mi@naver.com (S.-M.Y.); wso219@naver.com (M.-Y.S.); 2 Department of Agricultural Biology, National Institute of Agricultural Science, Rural Development Administration, Wanju 55365, Korea; ji35879@naver.com (S.-D.J.); sonjg@korea.kr (J.-G.S.)

**Keywords:** hepatic fibrosis, hepatocellular carcinoma, TGF-β, STAT3, steamed and freeze-dried mature silkworm larval powder, Wistar rat

## Abstract

Hepatocellular carcinoma (HCC) is the leading cause of cancer-related deaths worldwide and the majority of HCC patients occur with a background of hepatic fibrosis and cirrhosis. We have previously reported the hepatoprotective effects of steamed and freeze-dried mature silkworm larval powder (SMSP) in a chronic ethanol-treated rat model. Here, we assessed the anti-fibrotic and anti-carcinogenic effects of SMSP on diethylnitrosamine (DEN)-treated rats. Wistar rats were intraperitoneally injected with DEN once a week for 12 or 16 weeks with or without SMSP administration (0.1 and 1 g/kg). SMSP administration significantly attenuated tumor foci formation and proliferation in the livers of the rats treated with DEN for 16 weeks. SMSP administration also inhibited hepatic fibrosis by decreasing the levels of collagen fiber and the expression of pro-collagen I and alpha-smooth muscle actin (α-SMA). Moreover, SMSP supplementation improved the major parameters of fibrosis such as transforming growth factor-β (TGF-β), connective tissue growth factor (CTGF), tumor necrosis factor-alpha (TNF-α), plasminogen activator inhibitor-1 (PAI-1), and collagen type I (Col1A1) in the livers from the rats treated with DEN for 16 weeks. As s possible mechanisms, we investigated the effects of SMSP on the TGF-β and signal transducer and activator of transcription 3 (STAT3)-mediated signaling cascades, which are known to promote hepatic fibrosis. We found that SMSP treatment inhibited the activation of TGF-β and the phosphorylation of STAT3 pathway in DEN-treated rats. Moreover, SMSP administration suppressed the expressions of the target genes of TGF-β and STAT3 induced by DEN treatment. Our findings provide experimental evidences that SMSP administration has inhibitory effects of hepatic fibrosis and HCC induced by DEN In Vivo and could be a promising strategy for the prevention or treatment of hepatic fibrosis and hepatocellular carcinogenesis.

## 1. Introduction

Liver diseases represent a serious world public health problem [1]. In particular, hepatic fibrosis is a reversible wound-healing response to either acute or chronic liver injury including infection with hepatitis B and C, disorders in metabolism, and toxic insults [2,3]. During liver fibrogenesis, hepatic stellate cells (HSCs) are the major cell type responsible for abnormal matrix deposition [4]. In chronic liver disease, HSCs activate fibrogenic myofibroblasts, and increase proliferation, and change morphology expressing alpha-smooth muscle actin (α-SMA) and collagens [5,6]. Progression of disease with sustained fibrosis leads to cirrhosis, which may develop hepatocellular carcinoma (HCC) [2,7]. However, hepatic fibrosis is commonly asymptomatic and no drug has yet been approved as an effective anti-fibrotic agent [1,2].

Transforming growth factor-β (TGF-β) has been studied as a key mediator to activate fibrosis in all organs including the liver [8]. In fibrotic diseases, the levels of TGF-β and TGF-β target genes are up-regulated [9]. Likewise, liver fibrosis is accompanied by up-regulated levels of TGF-β isoforms [10]. The activated TGF-β binds to its receptors and then downstream signaling is mediated by Smad2 and 3 [11]. Phosphorylated Smad2/3 from heteromeric complexes with Smad4 translocate to the nucleus, where they promote TGF-β target genes such as plasminogen activator inhibitor-1 (PAI-1), collagen type I (Col1A1), and connective tissue growth factor (CTGF) [11]. However, inhibition of these pathways has not been reported to completely improve the fibrotic symptoms of TGF-β, which means that other pathways may be present to regulate the inhibitory effects of fibrosis [12]. Identification of other molecules to inhibit fibrogenesis might provide potential targeted therapies for fibrosis.

Signal transducer and activator of transcription 3 (STAT3) regulates cell growth, proliferation, differentiation, and migration [13]. STAT3 signaling pathway has been studied in the pathogenesis of liver diseases [14]. STAT3 is activated by interleukin-6 (IL-6) which has been identified as a key regulator of the STAT3 signaling pathways [11]. The phosphorylation of STAT3 at Tyr705 leads to dimerization, translocation to nucleus, recognition of STAT3 specific DNA-binding elements, and up-regulation of STAT3 target genes [15]. In particular, STAT3 is known to be activated as a result of the inflammatory response that represents the first step of wound healing [16]. Because fibrosis can be considered as the process of wound healing after injury [17], several studies have focused on the relationship between fibrosis and the STAT3 signaling pathway [12,18,19]. The various upstream of STAT3 have been studied such as janus kinase 2 (JAK2), protein kinase B (Akt), epidermal growth factor receptor (EGFR) and Src [9,20,21]. However, recent studies have shown that TGFβ and STAT3 interactions regulate the process of fibrosis. Thus, inhibition of both TGF-β and STAT3 signaling pathways can lead to therapeutic effects on fibrosis.

Entomophagy, the practice of eating insects, is traditionally consumed in many countries. Generally, insects contains high nutrients and good source of fat, proteins, minerals, and vitamins [22]. The silkworm, *Bombyx mori*, has been used for silk production since ancient times [23], but it is emerging as a material with beneficial health effects [24]. In particular, the silkworm (*Bombyx mori*) contains a lot of crude proteins, vitamins, minerals, and n-3 fatty acids [24], hence it has been reported to therapeutic agents for the treatment of Parkinsons’s disease [25], hyperlipidemia, hyperglycemia [26], and liver diseases [27]. We previously reported that steamed and freeze-dried mature silkworm larval powder (SMSP) reduced liver injury-related biomarkers and pro-inflammatory cytokines in acute treatment with diethylnitrosamine (DEN) in mice [28]. A more recent study has demonstrated that SMSP prevents hepatic steatosis and fibrosis in ethanol-treated rats [27]. Nevertheless, the protective role of SMSP against hepatic fibrosis and hepatocellular carcinogenesis has not yet been reported in chronic treatment with DEN in rats. Therefore, we investigated the beneficial effects of SMSP on DEN-induced hepatic fibrosis and HCC in a rat model and elucidated the underlying molecular mechanisms.

## 2. Materials and Methods

### 2.1. Reagents and Antibodies

DEN was purchased from Sigma (St. Louis, MO, USA). Antibodies for transforming growth factor-beta receptor (TGF-βR) I, TGF-βRII, phospho-EGFR, and β-actin were purchased from Santa Cruz Biotechnology (Santa Cruz, CA, USA). Antibodies for phospho-Smad2, phospho-Smad3, Smad2/3, phospho-STAT3, STAT3, phospho-Akt, Akt, phospho-Jak2, Jak2, phospho-Src, and Src were purchased from Cell Signaling Technology (Danvers, MA, USA). Antibodies for proliferating cell nuclear antigen (PCNA), antigen Ki-67 (Ki67), glutathione S-transferase pi (GST-pi) and α-SMA were purchased from Abcam (Cambridge, UK).

### 2.2. Animal Experiment

Five-week-old male Wistar rats in the experiment were purchased from Orient bio (Gyeonggi-do, Korea). All animals were housed in an experimental room at 24 °C with 12 h light/dark cycle. The animals were handled at accredited animal facilities in accordance with the Institutional Animal Care and Use Committee (IACUC) of the CHA University Animal Center (reference number: IACUC 170061). The animals were allowed an acclimatization period of one week. Thereafter, healthy six-week-old Wistar rats were randomly assigned into four groups (*n* = 10/group), which were each assigned to one of four experimental diets: American institute of nutrition (AIN)-76A (Normal and DEN group), and AIN-76A supplemented with SMSP 0.1 or 1 g/kg, respectively. AIN-76A and SMSP containing AIN-76A diets were purchased from DBL (Umsung, Korea). The rats were subjected to the experimental diet for 12 and 16 weeks, during which time all animals were allowed ad libitum access to diet and water. Male Wistar rats received weekly intraperitoneal injections of DEN (50 mg/kg) or phosphate-buffered saline (PBS) for 12 and 16 weeks. At the end of the experiment, the rats were sacrificed at 12 and 16 weeks. Blood was collected from the abdominal aorta into a heparin tube. Serum was subsequently obtained by centrifuging the blood at 3000 rpm for 15 min at 4 °C. The livers were excised, rinsed with PBS, and weighed, and liver foci were counted and measured, and the portion of each liver was fixed in 10% formalin for further analysis. Serum and liver samples were stored at −80 °C until analysis.

### 2.3. Preparation of Steamed and Freeze-Dried Mature Silkworm Larval Powder

SMSP was made as previously described [29]. Briefly, live mature larvae of *Bombyx mori* white-jade cocoon strain were immediately steamed for 130 min at 100 °C using an electric pressure-free cooking machine (KumSeong Ltd., Boocheon, Korea) and freeze-dried using freeze-drier (FDT-8612, Operon Ltd., Kimpo, Korea) for 24 h. Then, larvae were grinded using a disk mill (Disk Mill01, Korean Pulverizing Machinery Co. Ltd., Incheon, Korea) and a hammer mill (HM001, Korean Pulverizing Machinery Co. Ltd., Incheon, Korea). The length of the SMSP particles was shorter than 0.1 mm. SMSP was stored at −50 °C and then used for formulating the diet for rats.

### 2.4. Biochemical Analysis

The serum levels of aspartate aminotransferase (AST), alkaline phosphatase (ALP), alanine aminotransferase (ALT), bilirubin, lactate dehydrogenase (LDH), albumin, glucose, triglyceride, total cholesterol, and low density lipoprotein (LDL) cholesterol were determined using a Hitachi automatic analyzer 7600-210 (Hitachi High-Technologies Corporation, Tokyo, Japan). The serum concentration of tumor necrosis factor-alpha (TNF-α), interleukin-1β (IL-1β) and TGF-β1 were measured by using a commercially available rat ELISA kit (R&D Systems, Minneapolis, MN, USA) following manufacturer’s instruction.

### 2.5. Histology and Immunohistochemistry

Formalin-fixed liver samples were embedded in paraffin, sliced at 5 µm, followed by sectioning and hematoxylin and eosin (H and E) or Masson’s trichrome staining by standard procedures. Immunohistochemistry (IHC) was conducted by using Vectastain ABC kit (Vectastain, PK6101 and PK6102). Briefly, sections were deparaffinized, hydrated and blocked with 3% hydrogen peroxide for 15 min. Then, specimens were subjected to antigen retrieval by immersing in 0.01 M boiling citrate buffer and heating in a microwave oven for 5 min and cooling down at room temperature. After blocking by blocking solution for 1 h, the sections were incubated 2 h at room temperature with primary antibodies against PCNA (1:20,000, ab29, Abcam, Cambridge, UK) or Ki67 (1:250, ab15580, Abcam, Cambridge, UK). After incubation, the slices were washed three times for 5 min with PBS. Then, the slices were incubated in biotinylated anti-mouse or anti-rabbit secondary antibody for 30 min at room temperature. Slides were then rinsed and incubated with avidin-biotinylated horseradish peroxidase (HRP) complex for 30 min at 37 °C. Slides were washed three times for 5 min, and PCNA or Ki67 was visualized by incubating for 30 sec in a solution containing 3,3′-diaminobenzidine tetrahydrochloride (DAB; Quanto, Thermo scientific, Waltham, MA, USA). Hematoxylin was used as a nuclear counterstain in tissue sections. Stained slides were dehydrated through a graded series of alcohol washes and mounted using cover slides.

### 2.6. RNA Isolation and Gene Expression Analysis

Total mRNA was isolated from the rat livers using Trizol reagent (Invitrogen, Carlsbad, CA, USA) and cDNA was synthesized using SuperScript^®^ II Reverse Transcriptase kit (Invitrogen, Carlsbad, CA, USA) according to the manufacturer’s instructions. The mRNA levels were analyzed by quantitative real-time polymerase chain reaction (qRT-PCR) and reverse transcription PCR (RT-PCR). The qRT-PCR was assessed as previously reported [30] and was performed on a ViiA™ 7 real-time PCR system (Life Technologies Corporation, Carlsbad, CA, USA) using Luna universal qPCR master mix (New England Biolabs, Beverly, MA, USA). RT-PCR was performed as previously reported [31] and was analyzed for 35 cycles at 94 °C for 20 s, 60 °C for 30 s, and 72 °C for 45 s; 18S ribosomal RNA (18s rRNA) were used as an internal control. The primer sets for qRT-PCR and RT-PCR are listed in Table 1.

### 2.7. Western Blot Analysis

Liver tissues were homogenized with ice-cold cell lysis buffer containing protease inhibitor (Roche Applied Science, Mannheim, Germany) and samples were incubated on ice with frequent vortexing for 5 min and centrifuged for 15 min at 13,000 rpm. The protein concentration of each supernatant was quantified using Pierce^TM^ BCA protein assay kit (Thermo Fisher Scientific, Waltham, MA, USA) in accordance with the manufacturer’s instructions. The proteins were loaded onto a 10% sodium dodecyl sulfate polyacrylamide gel electrophoresis (SDS-PAGE), and transferred to polyvinylidene fluoride membranes (Millipore, Burlington, MA, USA). After transfer, membranes were blocked with 3% bovine serum albumin (BSA) in Tris-buffered saline with 0.05% Tween 20 (TBS-T) and probed with the specified primary antibodies (diluted 1:1000) overnight at 4 °C. The membranes were washed and incubated with the appropriate secondary antibodies in TBS-T for 40 min. The blots were then developed using an enhanced chemiluminescence system (Thermo Fisher Scientific, Waltham, MA, USA).

### 2.8. Statistical Analysis

All data are expressed as means ± standard deviation (SD). Each experiment was performed a minimum of three times. Statistical analysis was performed using one-way analysis of variance (ANOVA). Statistical significance was accepted at *p* < 0.05.

## 3. Results

### 3.1. Steamed and Freeze-Dried Mature Silkworm Larval Powder (SMSP) Alleviates Liver Injury and Foci Formation in Diethylnitrosamine (DEN)-Treated Rats

**Figure 1:**
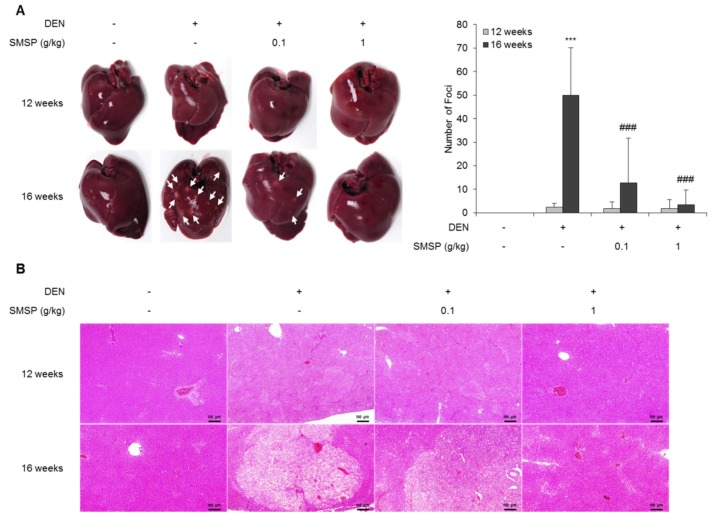
Steamed and freeze-dried mature silkworm larval powder (SMSP) decreases diethylnitrosamine (DEN)-induced foci formation. (**A**) Representative picture of livers. The number of foci (white arrows) was counted. (**B**) Representative image of hematoxylin and eosin (H and E)-stained sections of liver (scale bar = 100 µm). Data are the mean ± standard deviation (SD, *n* = 10). Statistical significance was analyzed by analysis of variance (ANOVA). *** *p* < 0.001 compared to control; ### *p* < 0.001 compared to the DEN group.

**Figure 2:**
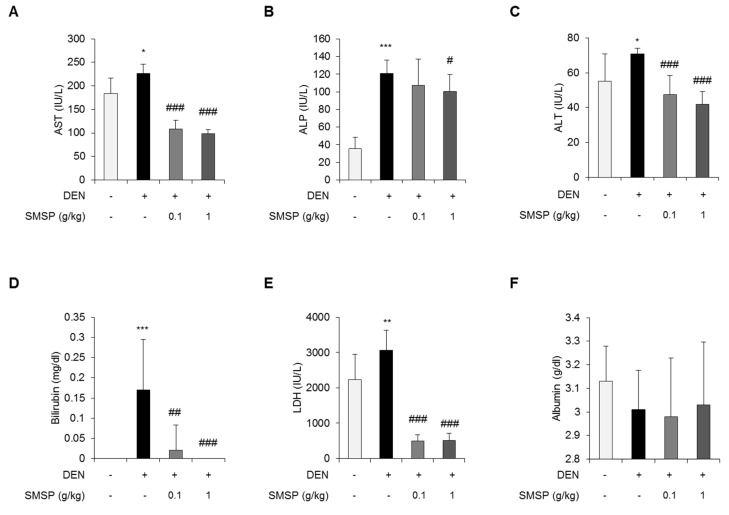
SMSP attenuates liver injury in rats treated with DEN for 16 weeks. Serum levels of (**A**) aspartate aminotransferase (AST), (**B**) Serum alkaline phosphatase (ALP), (**C**) alanine aminotransferase (ALT), (**D**) bilirubin, (**E**) lactate dehydrogenase (LDH), and (**F**) Albumin. Data are the mean ± SD (*n* = 10). Statistical significance was analyzed by ANOVA. * *p* < 0.05, ** *p* < 0.01, and *** *p* < 0.001 compared to control; # *p* < 0.05, ## *p* < 0.01, and ### *p* < 0.001 compared to the DEN group.

In order to prove the hypothesis that SMSP would improve liver injury and hepatocellular carcinogenesis, we evaluated the effect of SMSP in DEN-treated rats. We treated DEN-injured rats with SMSP by way of a dietary pellet of either 0.1 or 1 g/kg (*n* = 10 for each dose). Repeated injections of DEN (50 mg/kg weekly) in rats causes progressive liver fibrosis followed by HCC. Because DEN is mainly metabolized by CYP2E1 and high CYP2E1 activity is correlated with hepatic fibrosis, we investigated the hepatic expression of CYP2E1 mRNA in DEN-treated rats. As a result, treatment with DEN for 16 weeks significantly induced the hepatic expression of CYP2E1 mRNA compared with control rats (Figure S1). DEN injury for 12 weeks caused mimic foci formation (Figure 1A). After 16 weeks of DEN injection, the animals exhibited foci formation (Figure 1A). In contrast, livers from rats treated with DEN and SMSP had less foci formation (Figure 1A). However, changes of food consumption or body weight were not observed in rats supplemented by SMSP (0.1 and 1 g/kg) for 16 weeks (Figure S2). Further, histologic examination of liver sections stained by H and E revealed less severe foci formation in SMSP-fed rats, and a dose–response relationship was observed (Figure 1B). Although hepatic fibrosis or HCC was not observed in rats treated with DEN for 12 weeks, the serum levels of ALT, AST, ALP, and bilirubin were significantly elevated (Figure S2). A hepatoprotective effect of SMSP was observed in rats treated with DEN for 12 weeks, but there was no statistical significance (Figure S2). However, DEN-induced liver injury was significantly improved in the rats that received 0.1 or 1 g/kg SMSP for 16 weeks. The serum levels of AST, ALP, ALT, bilirubin, and LDH were reduced in rats treated with both DEN and SMSP (Figure 2A–E). However, the serum levels of albumin were not changed (Figure 2F). Since lipid metabolism is one of the important functions of the liver [32], we investigated the factors involved in lipid metabolism. As a result, we observed that SMSP administration can improve the serum levels of glucose, triglyceride, total cholesterol, and LDL cholesterol in rats treated with DEN for 16 weeks (Figure S4). These results suggest that SMSP may prevent liver damage and foci formation induced by DEN in rats.

### 3.2. SMSP Prevents Hepatocellular Carcinogenesis in DEN-Treated Rats

**Figure 3:**
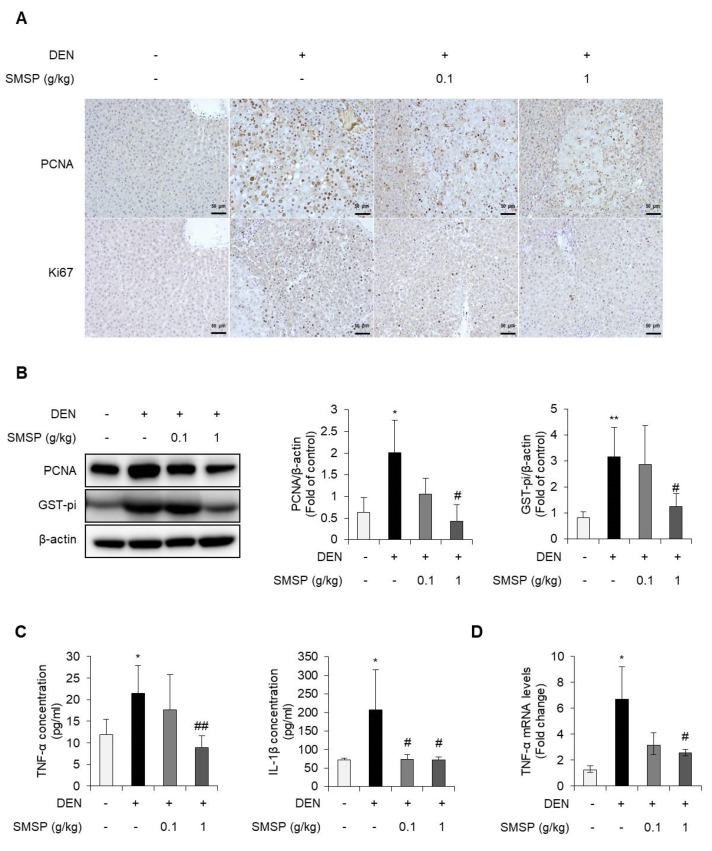
SMSP decreases hepatic proliferation and inflammation in rats treated with DEN for 16 weeks. (**A**) Representative immunohistochemical pictures of proliferating cell nuclear antigen (PCNA) or Ki67 in rats. (**B**) Hepatic PCNA and GST-pi protein expressions were determined by Western blot analysis. Quantification of each protein expression relative to control group was exhibited. Data shown as mean ± SD. (**C**) Serum levels of pro-inflammatory cytokines, tumor necrosis factor-alpha (TNF-α) and interleukin-1β (IL-1β) were measured by enzyme-linked immunosorbent assay (ELISA). (**D**) The hepatic expression of TNF-α was measured by quantitative real-time polymerase chain reaction (qRT-PCR). Statistical significance was analyzed by ANOVA. * *p* < 0.05 and ** *p* < 0.01 compared to control; # *p* < 0.05 and ## *p* < 0.01 compared to the DEN group.

Several studies have revealed that PCNA is an important marker of cell division and expresses malignance, vascular infiltration, distatnt metastasis, and survival [33]. Ki67 is the hallmark protein involved in cell proliferation and its expression is a great marker for carcinoma of the breast, prostate, lymphoma, and liver [33]. Administration of SMSP exhibited a significant reduction in foci number and volume after 16 weeks compared with the DEN group in this study (Figure 1). Therefore, we next investigated the expressions of PCNA and Ki67 as tumor cell proliferative markers in the liver tissues. In the immunohistochemical analyses, the liver sections from the DEN group exhibited greater tumor cell proliferation compared with SMSP-treated specimens. In addition, the PCNA- or Ki67-positive immunostained cells were decreased by SMSP administration for 16 weeks. These findings indicated that the treatment with SMSP for 16 weeks reduced abnormal proliferation of liver cells induced by DEN (Figure 3A). Western blot analyses showed the decreased expressions of PCNA and GST-pi in the liver tissues from SMSP-fed rats compared with DEN-treated rats (Figure 3B). Chronic inflammation has been known to be associated with persistent liver injury, leading to fibrosis and HCC [34]. Based on this rationale, we analyzed the serum levels of TNF-α and IL-1β, representative pro-inflammatory cytokines. As shown in Figure 3C, the levels of TNF-α and IL-1β were significantly increased in DEN-treated rats while SMSP treatment ameliorated the serum levels of TNF-α and IL-1β. Moreover, the hepatic mRNA expression of TNF-α was significantly downregulated by SMSP administration (Figure 3D). These results indicate that SMSP treatment attenuates inflammation and proliferation, thereby inhibiting hepatocellular carcinogenesis.

### 3.3. SMSP Attenuates Hepatic Fibrosis in DEN-Treated Rats

**Figure 4:**
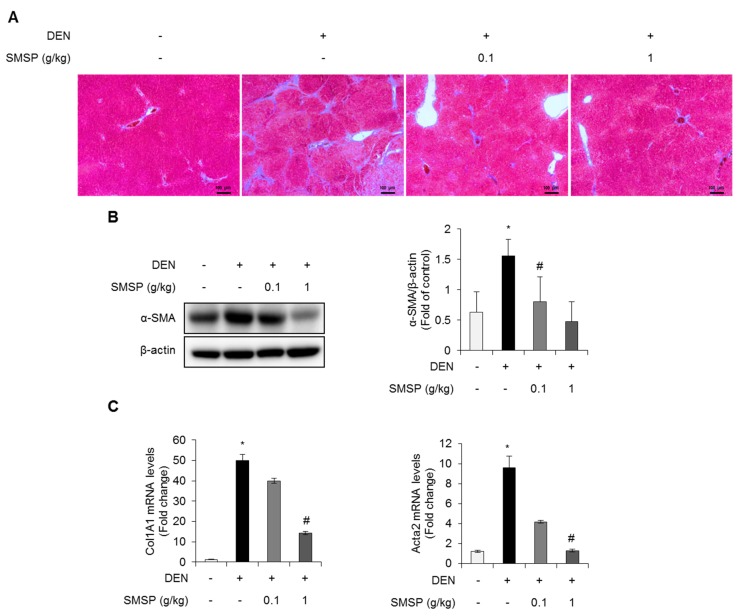
SMSP attenuates hepatic fibrosis in rats treated with DEN for 16 weeks. (**A**) Histopathological sections of the liver were stained with Masson’s trichrome method (scale bar = 100 µm). (**B**) Hepatic alpha-smooth muscle actin (α-SMA) protein expression was determined by Western blotting. Quantification of α-SMA protein relative to control group was exhibited. (**C**) Hepatic *Col1A1* and *Acta2* mRNA levels were measured by qRT-PCR. All mRNA levels were normalized relative to *Rn18S*. Results are presented as mean ± SD. Statistical significance was analyzed by ANOVA. * *p* < 0.05 compared to control; # *p* < 0.05 compared to the DEN group.

To explore the further effects of SMSP on hepatocellular carcinogenesis, we next investigated the fibrotic changes in liver tissues from this animal model. Administration of SMSP for 16 weeks significantly inhibited the hepatic fibrogenesis induced by DEN in rats. Histologic examination of liver sections stained by Masson’s trichrome method showed the noticeable blue staining of collagen fiber with the destruction of liver structure in rats treated with DEN. However, SMSP administration significantly decreased the hepatic collagen fiber in DEN-treated rats (Figure 4A). During chronic liver injury, extracellular membrane (ECM) develops hepatic fibrosis and the most important structural ECM components in the liver is collagen [2]. In addition, a characteristic marker of mesenchymal cells is known as α-SMA [2]. To confirm the antifibrotic effect of SMSP treatment on DEN-induced hepatic fibrosis, we performed the Western blot analysis to determine the protein expression of α-SMA. Increased expression of α-SMA induced by DEN injection was significantly reduced in SMSP-fed groups (Figure 4B). qRT-PCR was also employed to detect mRNA expressions of fibrosis markers, such as alpha-1 type I collagen (Col1A1) and alpha-smooth muscle actin (Acta2), which encode major components of Type I collagen and α-SMA, respectively. Up-regulated expressions of Col1A1 and Acta2 mRNA in DEN-treated group were dose-dependently inhibited in SMSP-treated groups. (Figure 4C). These findings implicate the inhibitory effects of SMSP administration on hepatic fibrosis induced by chronic DEN treatment.

### 3.4. SMSP Inhibits Transforming Growth Factor-β (TGF-β) Signaling Pathway

**Figure 5:**
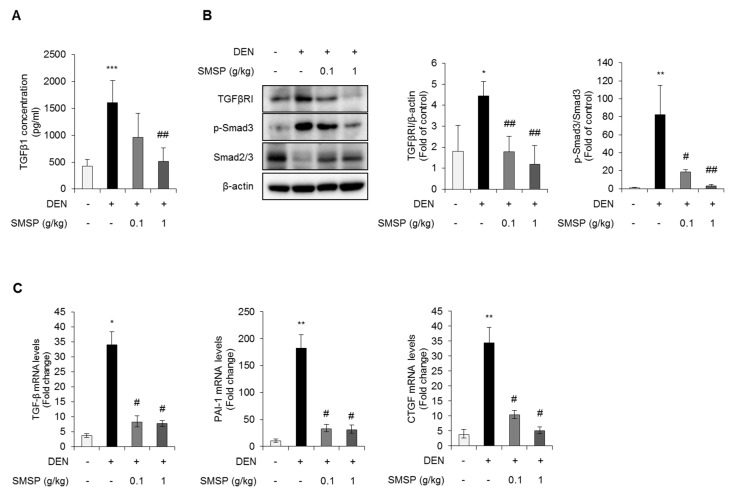
SMSP decreases transforming growth factor-β (TGF-β)-mediated signaling cascades in rats treated with DEN for 16 weeks. (**A**) Serum levels of TGF-β1 were measured by ELISA. (**B**) The protein expression of TGF-βR, phopho-Smad3, and Smad3 were analyzed by Western blotting and relative protein expressions were quantified. (**C**) The mRNA expressions of *TGF-β*, *PAI-1*, and *CTGF* were evaluated by qRT-PCR analysis. Results are presented as the mean ± SD. Statistical significance was analyzed by ANOVA. * *p* < 0.05, ** *p* < 0.01, and *** *p* < 0.001 compared to control; # *p* < 0.05 and ## *p* < 0.01 compared to the DEN group.

Since TGF-β plays a crucial role in the development of fibrosis [35], we evaluated the inhibitory effect of SMSP on the TGF-β signaling pathway in DEN-induced hepatic fibrosis. As a result, serum levels of TGF-β were completely decreased by SMSP administration for 16 weeks (Figure 5A). The increased hepatic expressions of TGF-βRI and phospho-Smad3 induced by DEN treatment were significantly downregulated in rats treated with SMSP (Figure 5B). However, the expressions of TGF-βRII and phospho-Smad2 were not changed (Figure S5A). Moreover, the increased mRNA expression level of TGF-β, PAI-1, and CTGF induced by DEN treatment were also suppressed by SMSP administration (Figure 5C). These results indicate that SMSP administration inhibits the TGF-β signaling pathway.

### 3.5. SMSP Inhibits Signal Transducer and Activator of Transcription 3 (STAT3)-Mediated Signaling Pathways in the Liver

**Figure 6:**
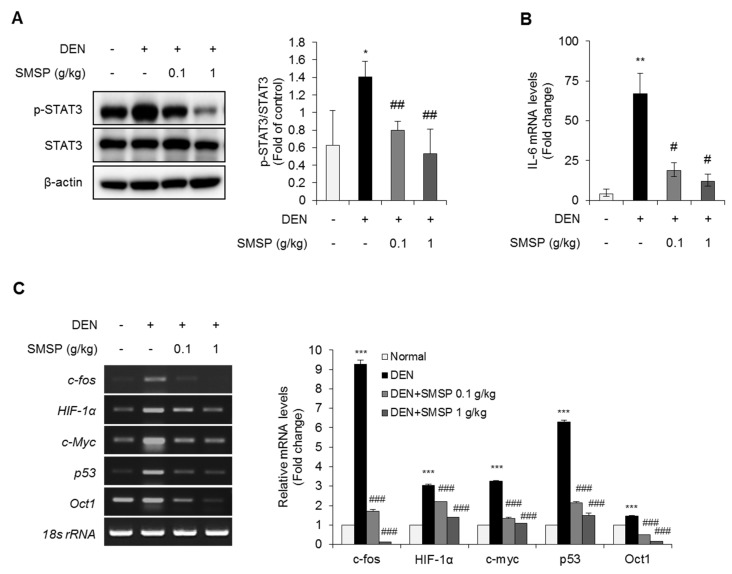
SMSP inhibits signal transducer and activator of transcription 3 (STAT3) phosphorylation and related signaling in rats treated with DEN for 16 weeks. (**A**) Hepatic protein levels of p-STAT3 and STAT3 were determined by immunoblotting. The level of p-STAT3 relative to control group was quantified. (**B**) Hepatic *IL-6* mRNA level was measured by qRT-PCR. *IL-6* mRNA levels were normalized relative to *Rn18S*. (**C**) Hepatic mRNA levels of STAT3-target genes, *c-fos*, *HIF-1α*, *c-Myc*, *p53*, and *Oct1* were measured by RT-PCR. Quantification of mRNA expressions relative to control group was exhibited. All mRNA levels were normalized relative to *Rn18S*. Results are presented as the mean ± SD. Statistical significance was analyzed by ANOVA. * *p* < 0.05, ** *p* < 0.01, and *** *p* < 0.001 compared to control; # *p* < 0.05, ## *p* < 0.01, and ### *p* < 0.001 compared to the DEN group.

Fibrosis occurs when the liver is continuously and repeatedly damaged [36]. Recent studies emphasized the role of STAT3 in fibrogenesis [37]. The activation of STAT3 pathway has been reported to play an important role during tissue restoration after tissue damage [38]. To investigate the underlying molecular mechanisms, we examined the effect of SMSP supplementation on the STAT3 signaling pathway. As shown in Figure 6A, DEN treatment induced the hepatic phosphorylation of STAT3 more than 2 times that of the control group. SMSP treatment decreased the phosphorylation of STAT3 dose-dependently. Furthermore, we confirmed the upstream regulators of STAT3, however, treatment with SMSP had no effect on the regulation of Jak, Akt, EGFR, and Src (Figure S5B). Next, we performed qRT-PCR to examine the mRNA expression of IL-6 known as a classical activator of the phosphoylation of STAT3. The expression of IL-6 mRNA was significantly induced in the livers of DEN-treated rats compared with the control group. However, SMSP administration to DEN-treated rats reduced the hepatic expression of IL-6 mRNA remarkably. We further investigated the effect of SMSP on DEN-mediated STAT3 signaling in the liver. Treatment with DEN significantly induced the hepatic expression levels of target genes, such as *c-fos, HIF-1α, c-Myc, p53* and *Oct1*, while SMSP administration significantly reduced the hepatic expressions of *c-fos, HIF-1α, c-Myc, p53* and *Oct1* (Figure 6C). These findings suggest that SMSP is effective in positively regulating DEN-induced STAT3 signaling pathway.

## 4. Discussion

Diethylnitrosamine (DEN), also known as *N*-nitrosodiethylamine, is one of the most important environmental carcinogens to cause tumors in various organs [39]. Specifically in the liver, DEN is hydroxylated by the cytochrome P450 2E1 (CYP2E1), resulting in DNA-adducts through an alkylation mechanism, which is a critical process in DEN-induced HCC [39]. In this study, 50 mg/kg of DEN was repeatedly injected once a week for 16 weeks and this dose was enough to induce fibrosis followed by HCC in Wistar rats [40]. In the current study, we elucidated the hepatoprotective effect of SMSP administration in DEN-induced hepatic fibrosis and HCC in a rat model, likely through suppression of the TGF-β/STAT3 signaling pathway.

Because SMSP contains diverse functional materials, such as silk fibroin proteins, flavonoids, vitamins, fatty acids, polyphenols, and essential minerals [24], it has been studied to improve liver functions [26,28]. In acute and sub-acute toxicity studies for silkworm powder, it has been reported that the administration of 2 g/kg silkworm powder showed no mortality and no body weight changes in Wistar rats [41]. In addition, we confirmed that up to 10 g/kg of silkworm powder has no toxicity according to a study conducted by an authorized research center (data not shown). In the present study, rats were fed 0.1 and 1 g/kg, corresponding to an intake of approximately 0.973 and 9.73 g/60 kg adult/day, respectively, when calculated on the basis of normalization to body surface area as recommended by Reagan-Shaw et al. [42]. In this regards, we found that food consumption or body weight were not changed in rats supplemented by SMSP (0.1 and 1 g/kg) for 16 weeks. In this study, we administered SMSP to rats for 16 weeks, which is approximately equivalent to 12 years of a human lifetime [43]. This suggests that a steady intake of SMSP can prevent hepatocellular carcinogenesis.

Generally, mature silkworm after the 3rd day of the 5th instar was difficult to ingest due to enlarged silk glands. To solve this problem, Ji et al. developed technique to make mature silkworm larvae edible by steaming silkworm 130 min at 100 °C before being freeze-dried and ground [29]. SMSP has been reported to contain 70% crude protein, mainly fibroin and sericin, which repeated structures of amino acids, serine, glycine, and alanine. A large amount of amino acids diet is known to improve health [24]. Liver is an important organ for amino acid metabolism [44], and abundant amino acids are present [45]. Some amino acids have been demonstrated to have In Vivo potential pharmacological properties for hepatic steatosis, fibrosis, and necrosis [46]. Alanine was reported to prevent rats from hepatocyte necrosis and diet-induced obesity [47]. Glycine was also effective for the inhibition of carbon tetrachloride-induced hepatic fibrosis and alcohol-induced liver damage in rats [48,49]. Serine was reported to alleviate alcohol-induced fatty liver in mice and rats [50]. Besides flavonoids, polyphenols, vitamins, and minerals were also present in SMSP in considerable amounts [24], these constituents may have beneficial effects in the liver. Our findings showed that SMSP administration can improve the lipid metabolism as well as the liver damage, fibrosis, and hepatocellular carcinogenesis in DEN-treated rats. These hepatoprotective effects of SMSP are considered to be manifested by the aforementioned active ingredients of SMSP. The previous studies suggest that the health promotion effects of SMSP could be accomplished by the complex actions of several substances [24,51]. Therefore, the liver cancer preventive effect of SMSP is not thought to be caused by one particular ingredient of SMSP. For this reason, SMSP could be considered as a functional food that can prevent cancer or carcinogenesis rather than an anti-cancer drug. Previous studies in animals and humans have shown that the hydrolysate of fibroin, a major component of silk fibers, enhanced memory and cognition [52]. However, the effect of silk protein, major components of SMSP, on hepatocellular carcinogenesis has not been reported to date. Therefore, a more detailed mechanistic study should be conducted with functional components of SMSP such as silk proteins, peptides, or amino acids.

STAT3 is a cytoplasmic transcription factor that plays pivotal roles in cellular functions including proliferation, survival, migration, and differentiation [13]. In recent years, several studies reported that STAT3 plays a key role in the development of fibrosis of diverse organs [53]. Liver fibrosis is an incomplete recovery from hepatocyte injury [54]. During fibrogenesis, the ECM and excess matrix contraction are overproduced and liver architecture is disrupted [54]. Recent research suggests that activation of liver HSCs plays and important role in fibrogenesis via induction of ECM proteins [55]. In particular, STAT3 promotes fibrosis by inducing the production of the ECM, and IL-6 activates STAT3 in HSCs [38,56]. Hepatic fibrosis induced by carbon tetrachloride has been promoted in IL-6-deficient mice [57]. STAT3 inhibitor HJC0123 inhibited fibrotic markers, such as type I collagen and ECM protein fibronectin in HSCs [37] and another STAT3 inhibitor, S3I-201 has also shown amelioration of fibrosis in preclinical models [58,59]. Based on this evidence, we evaluated the efficacy of SMSP on the STAT3 signaling pathway in DEN-induced hepatic fibrosis animal model. We found that SMSP administration inhibited the activation of STAT3 pathway as well as the inhibition of STAT3 target genes. However, treatment with SMSP had no effect on the regulation of Jak, Akt, EGFR, and Src. These results demonstrated that SMSP administration inhibits the STAT3 signaling pathway induced by DEN through other pathways than the upstream kinases mentioned above in our study.

Recent studies have revealed that TGF-β also induced the STAT3 signaling pathway [12,60]. The TGF-β signaling pathway is crucial in the regulation of fibrosis development [8]. The TGF-β signal regulates cell proliferation, differentiation, tissue homeostasis, regeneration, and severe diseases in mammalian cells [61]. The TGF-β signaling pathway occurs through type I and type II receptors (TβRI and TβRII) [62]. Both receptors are similar trans-membrane serine/threonine kinases, but TβRI has a conserved Gly/Ser rich upstream [63]. After ligand engagement of TGF-β receptors, phosphorylation of TβRII leads to TβRI activation [62]. Activated TβRI phosphorylates Smad2/3 proteins, which translocate to the nucleus and induce target genes [11]. It has been reported that carbon tetrachloride-treated mice with heterozygous deletion of platelet TGF-β1 (PF4CreTgfb1^f/f^) showed decreased fibrosis and related signals including Smad2, pro-collagen, and α-SMA expressions [64]. In this study, we found that treatment of rats with SMSP inhibited TGF-β-mediated fibrotic markers, such as TGF-β, PAI-1, CTGF, TGF-βRI and phospho-Smad3 as well as the serum levels of TGF-β, which were induced by DEN treatment. However, there was no change in the expression of TGF-βRII and phospho-Smad2. Although Smad2 and Smad3 are known as the downstream signals of TGF-β, they are functionally distinct with different expression patterns [4]. Smad2 null mice die before birth, but Smad3 null mice survive until adulthood [65,66]. Expression of dominant negative Smad3, not Smad2 prevents TGF-β-mediated inhibition of adipocyte differentiation [67]. Furthermore, Uemura et al. have reported that Smad3 plays a key role in multiple important functions of transdifferentiated HSCs In Vitro [4]. Based on this evidence, the inhibitory effect of SMSP on phospho-Smad3, not Smad2, is not surprising.

In conclusion, the present study demonstrated that SMSP has protective effects against DEN-induced hepatic fibrosis and hepatocellular carcinogenesis in rats. We suggest that beneficial components in SMSP may have a favorable effect by decreasing inflammation, fibrosis, and HCC incidence. These beneficial effects are mediated by the inhibition of the TGF-β/STAT3 signaling pathway. Our findings provide experimental evidence on the molecular explanation of SMSP for the inhibition of hepatic fibrosis and HCC induced by DEN In Vivo. Taken together, SMSP could be a potential strategy for the prevention or treatment of hepatic fibrosis and hepatocellular carcinogenesis.

## Figures and Tables

**Table 1:** List of primers.

	Gene	Forward	Reverse	Size (bp)
qRT-PCR	TNF-α	ACTGAACTTCGGGGTGATCG	GCTTGGTGGTTTGCTACGAC	153
	Col1a1	CAACCTCAAGAAGTCCCTGC	AGGTGAATCGACTGTTGCCT	77
	Acta2	ACTGGGACGACATGGAAAAG	GCCACATACATGGCAGGGACATTG	172
	TGF-β	GCGGACTACTACGCCAAAGA	TGCTTCCCGAATGTCTGACG	129
	PAI-1	CGTCTTCCTCCACAGCCATT	GTTGGATTGTGCCGAACCAC	97
	CTGF	ACCCAACTATGATGCGAGCC	GCCCATCCCACAGGTCTTAG	77
	IL-6	TCCTACCCCAACTTCCAATGCTC	TTGGATGGTCTTGGTCCTTAGCC	79
	CYP2E1	AAACAGGGTAATGAGGCCCG	AGGCTGGCCTTTGGTCTTTT	78
	18s rRNA	GCAATTATTCCCCATGAACG	GGCCTCACTAAACCATCCAA	111
RT-PCR	c-Fos	TACTACCATTCCCCAGCCGA	GCGTATCTGTCAGCTCCCTC	409
	HIF-1α	GCCCCAGATTCAAGATCAGCC	ATTCATCAGTGGTGGCAGTTGCG	392
	c-Myc	ACTCGGTGCAGCCCTATTTC	GTAGCGACCGCAACATAGGA	187
	p53	CCCCTGAAGACTGGATAACTGT	GGTGGAAGCCATAGTTGCCT	348
	Oct1	GTCACATCTGTGTCCGGTGT	CACTAGCCCCACTGTGAAGG	195
	18s rRNA	CCCAACTTCTTAGAGGGACAAGT	TAGTCAAGTTCGACCGTCTTCTC	350

## Supplementary Materials

The following are available online at https://www.mdpi.com/2073-4409/9/3/568/s1, Figure S1: SMSP decreases the hepatic expression of CYP2E1 mRNA in rats treated with DEN for 16 weeks, Figure S2: Effect of SMSP on body weight and food intake in rats treated with DEN for 16 weeks, Figure S3: SMSP attenuates liver injury in rats treated with DEN for 12 weeks, Figure S4: SMSP attenuates liver injury in rats treated with DEN for 16 weeks, Figure S5: Effect of SMSP on TGF-β/STAT3 signaling in rats treated with DEN for 16 weeks.
