# Supplementary material for: Administration of Steamed and Freeze-Dried Mature Silkworm Larval Powder Prevents Hepatic Fibrosis and Hepatocellular Carcinogenesis by Blocking TGF-β/STAT3 Signaling Cascades in Rats
Source: Cells. 2020 Feb 28;9(3):568. doi: 10.3390/cells9030568 (PMC7140417; doi:10.3390/cells9030568)
Supplement: Supplementary file 1 [file cells-09-00568-s001.pdf]

cells

MDPI

Supplementary Figures

Administration of Steamed and Freeze-Dried Mature Silkworm Larval Powder Prevents Hepatic Fibrosis and Hepatocellular Carcinogenesis by Blocking TGF-β/STAT3 Signaling Cascades in Rats

Da-Young Lee [1], Sun-Mi Yun [1], Moon-Young Song [1], Sang-Deok Ji [2], Jong-Gon Son [2] and Eun-Hee Kim [1,*]

[1] College of Pharmacy and Institute of Pharmaceutical Sciences, CHA University, Seongnam 13488, Korea; angela8804@naver.com (D.-Y.L.); sun21mi@naver.com (S.-M.Y.); wso219@naver.com (M.-Y.S.)

[2] Department of Agricultural Biology, National Institute of Agricultural Science, Rural Development Administration, Wanju 55365, Korea; ji35879@naver.com (S.-D.J.); sonjg@korea.kr (J.-G.S.)

* Correspondence: ehkim@cha.ac.kr; Tel.: +82-31-881-7179

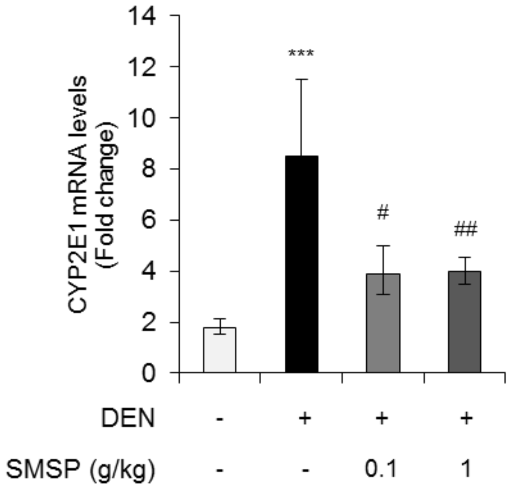

Figure S1. SMSP decreases the hepatic expression of *CYP2E1* mRNA in rats treated with DEN for 16 weeks. The hepatic expression of *CYP2E1* was measured by qRT-PCR and normalized relative to *Rn18S*. Statistical significance was analyzed by ANOVA. *** $P < 0.001$ compared to control; # $P < 0.05$ and ## $P < 0.01$ compared to DEN group.

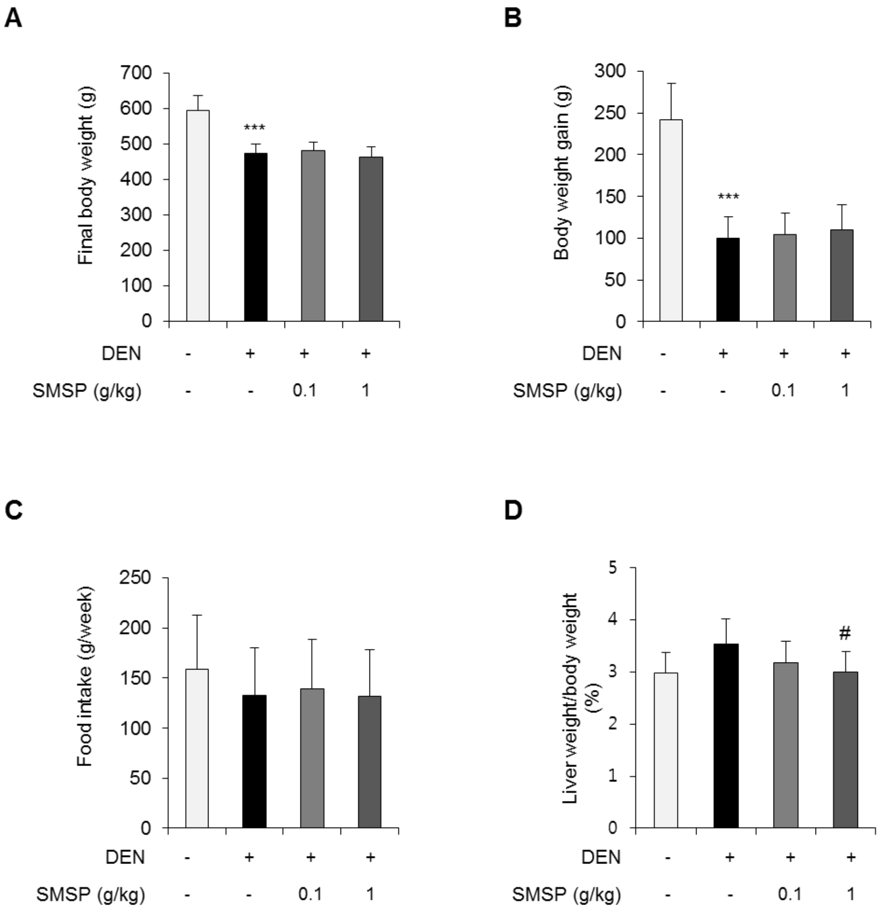

Figure S2. Effect of SMSP on body weight and food intake in rats treated with DEN for 16 weeks. (A) Body weight, (B) body weight gain, and (C) Food intake were recorded once a week. (D) The ratio of liver weight to body weight was calculated. Statistical significance was analyzed by ANOVA. *** $P < 0.001$ compared to control; # $P < 0.05$ compared to DEN group.

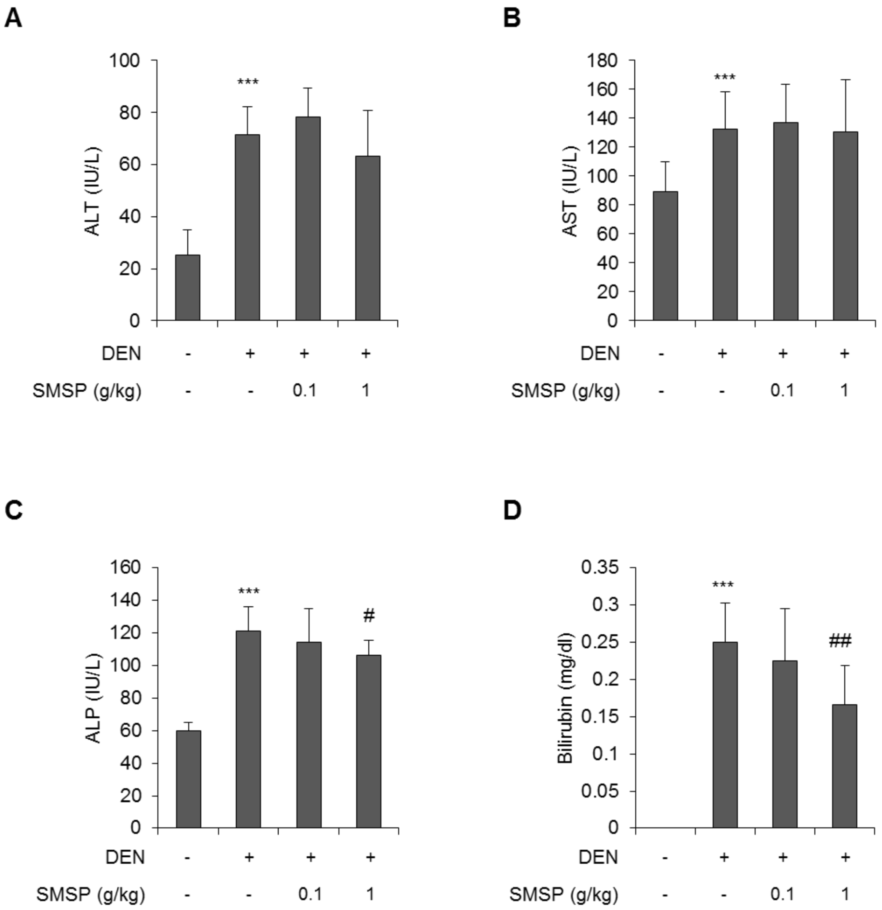

Figure S3. SMSP attenuates liver injury in rats treated with DEN for 12 weeks. Serum levels of (A) alanine aminotransferase (ALT), (B) aspartate aminotransferase (AST), (C) alkaline phosphatase (ALP), and (D) bilirubin were measured. Data are the mean ± SD (n = 10). Statistical significance was analyzed by ANOVA. *** $P < 0.001$ compared to control; # $P < 0.05$ and ## $P < 0.01$ compared to DEN group.

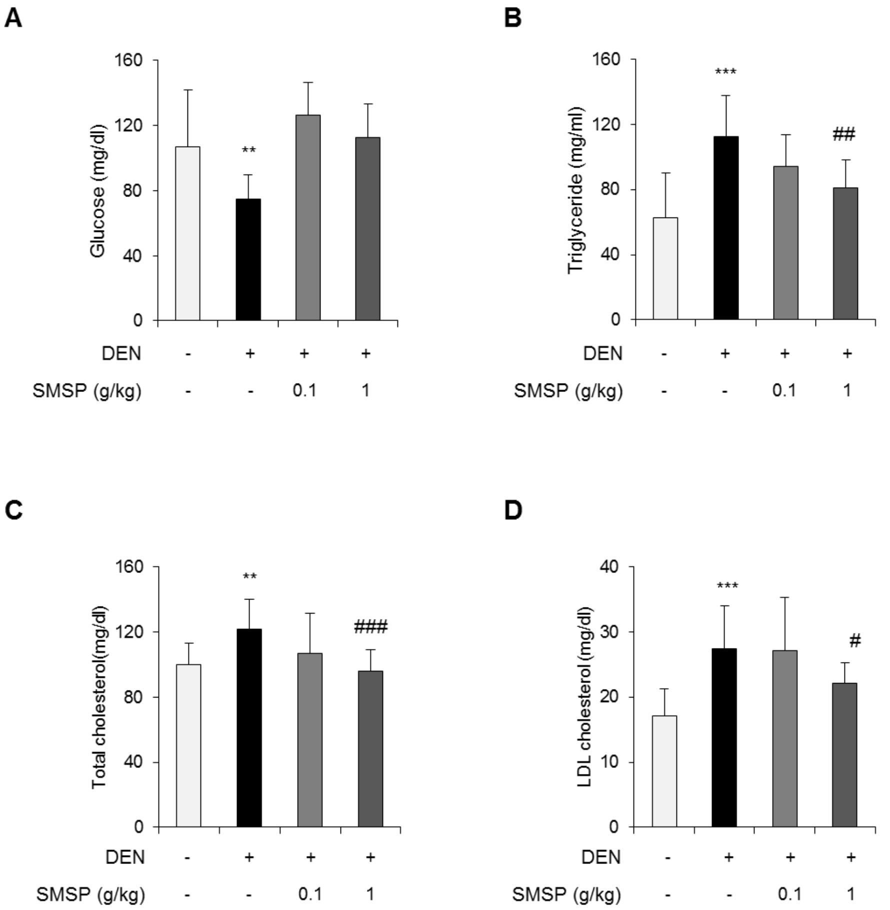

Figure S4. SMSP attenuates liver injury in rats treated with DEN for 16 weeks. Serum levels of (A) glucose, (B) triglyceride, (D) total cholesterol, and (D) LDL cholesterol were measured. Results are presented as mean ± SD (n = 10). Statistical significance was analyzed by ANOVA. ** $P < 0.01$ and *** $P < 0.001$ compared to control; # $P < 0.05$, ## $P < 0.01$ and ### $P < 0.001$ compared to DEN group.

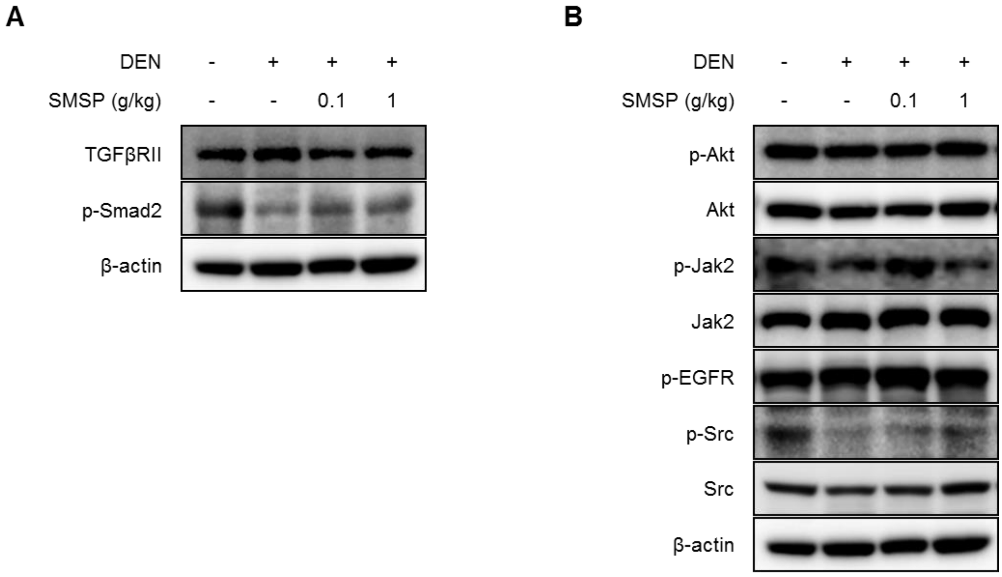

Figure S5. Effect of SMSP on TGF-β/STAT3 signaling in rats treated with DEN for 16 weeks. (A) The protein expressions of TGFβRII and p-Smad2 were analyzed by Western blotting. (B) The protein expressions of p-Akt, Akt, P-Jak2, Jak2, p-EGFR, p-Src, and Src were analyzed by Western blotting.
